# Utilizing Deep Eutectic Solvents in the Recycle, Recovery, Purification and Miscellaneous Uses of Rare Earth Elements

**DOI:** 10.3390/molecules29061356

**Published:** 2024-03-19

**Authors:** Francisco Jose Alguacil

**Affiliations:** Centro Nacional de Investigaciones Metalurgicas (CSIC), Avda. Gregorio del Amo 8, 28040 Madrid, Spain; fjalgua@cenim.csic.es

**Keywords:** deep eutectic solvents, rare earth elements, leaching, separation, purification

## Abstract

The boosted interest in using rare earth elements (REEs) in modern technologies has also increased the necessity of their recovery from various sources, including raw materials and wastes. Though hydrometallurgy plays a key role in these recovery processes, some drawbacks (apparent or not) of these processes (including the use of aggressive mineral acids, harmful extractants, and diluents, etc.) have led to the development of an environmental friendship subclass named solvometallurgy, in which non-aqueous solvents substituted to the aqueous media of the hydrometallurgical processing. Together with ionic liquids (ILs), the non-aqueous solvents chosen for these usages are the chemicals known as deep eutectic solvents (DEEs). The utilization of DEEs included the leaching of REEs from the different sources containing them and also in the separation-purification steps necessary for yielding these elements, normally oxides or salts, in the most purified form. This work reviewed the most recent literature (2023 year) about using deep eutectic solvents to recover REEs from various sources and coupling these two (DESs and REEs) to derive compounds to be used in other fields.

## 1. Introduction

Rare earth metals (REEs) are a group of elements of the periodic table consisting of seventeen chemical elements, including fifteen lanthanides plus scandium and yttrium. Moreover, this group is commonly divided into two subgroups: (i) light rare earth elements consisting of lanthanum, cerium, praseodymium, neodymium, promethium, samarium and europium, and (ii) heavy rare earth elements which include gadolinium, terbium, dysprosium, holmium, erbium, thulium, ytterbium, lutetium, and yttrium.

Since the XXI century, rare earth elements have gained extreme importance in industrialized countries. This is undoubtedly due to the development of the more sophisticated technologies that nowadays make life easier. With respect to their utility, these elements are key components in various markets, including magnets and permanent magnets, catalysts, metallurgy, phosphors, ceramics, glass, and polishing. Also, they are of the utmost importance in the military field and in the production of clean energy. As its name tends to indicate, rare earth elements are not evenly distributed along the world; in fact, REEs availability has China as the leader with 70% of worldwide production, followed by the USA (14.33%), Australia (6%), Myanmar (4%), Thailand (2.37%), Vietnam (1.43%), India (0.97%), Russia (0.87%), Madagascar (0.32%), and 0.03% for the rest of the world [1]. Reserves (in million MT) of these valuable metals are located in eight countries: China (44), Vietnam (22), Brazil and Russia (each 21), India (6.9), Australia (4.2), USA (2.3) and Greenland (1.5) [2].

Not all rare earths have the same price, with terbium and dysprosium being the ones with the higher price and lanthanum and cerium the cheapest. The current projects to recover these REEs are distributed along the world, but just one in Europe: Lovozersky (Russia, included in the top 30 REEs projects by estimated total value), and significantly, four advanced projects in Western Europe: one in Norway (Fen), two in Sweden (Norra Karr and Olserum) and one in Spain (Matamulas) [3].

The wide uses of REEs, combined with their relative scarcity and local accumulation of reserves, have led to their recovery from raw and secondary materials, which is gaining, day after day, a critical and strategic importance. Due to its operational characteristics (using low temperatures, the possibility of operating under pressure conditions, treatment of materials with low metal concentrations, the possibility of purification of complex materials and solutions, etc.), hydrometallurgy can be a major key for the recovery and purification of these strategic metals [4]. However, several environmental issues tend to consider traditional hydrometallurgy less environmentally friendly than the use of solvometallurgy in recovering valuable metals. In solvometallurgy, the utilization of aqueous medium is displaced by non-aqueous systems, even in the leaching step. However, despite the advantageous characteristics and performance, the implementation of non-aqueous solvents in metal leaching processes at a pilot or industrial scale is still very limited [5].

The utility of solvometallurgy has led to the development of investigations about the use of ionic liquids (ILs) and deep eutectic solvents (DESs) in the recovery of metals and the consideration of these two types of chemicals as the future of new green chemistry. However, as will be further mentioned, these chemicals are not as green as they appear. DESs and ILs are two different groups of chemicals with similar characteristics [6].

It is often considered that DESs can assist in the development of cleaner processes due to their properties: good thermal and chemical stability, low melting point, easy synthesis, low vapor pressure, and low or practically negligible toxicity, and probably one of the most important characteristics: tunability to meet specific applications [7,8,9]. Also, most of them are often biodegradable solvents, showing themselves to be candidates to be considered green solvents [10]. Against the consideration of the greenish character, it is indicated [11] that there is increased evidence about the non-greenness character of these chemicals precisely due to some of their characteristics, including instability, volatility, toxicity, flammability, and difficult regeneration.

In any case, besides its application in solvometallurgical processing, DESs are used in catalysis, electrolytic processes, and other processes, which are applicable to a series of process industries like food, pharmaceutical, cosmetics, oil, gas, etc. [9,12,13,14].

What are DESs? Further to the first approach to understanding the nature of this class of chemicals [15], the concept of DESs was developed and expanded. This deep nature was first explained by the preparation of different melts using metal chlorides (MCl_2_, M = Zn y/o Sn) with quaternary ammonium salts of formula [Me_3_NC_2_H_4_Y]Cl (Y = OH, Cl, OC(O)Me, OC(O)Ph) and abbreviated as liquid ionic Lewis acids [16,17].

As a general rule, DESs are obtained from the mixture of two or three substances with a given composition where the melting points of each of the individual components are higher than that of the mixture, consisting of the appropriate combination of hydrogen bond donors (HBDs) and hydrogen bond acceptors (HBA) [18,19]. Accordingly, DESs are cataloged into five types:

(i) TYPE I: the general formula for these DESs is Cat + X^−^x(MCln), where X^−^ and x refer to a Lewis base and the number of MCln in the DES unit, respectively.
(ii) TYPE II: these DESs are obtained from the same HBA, but the metallic chloride is hydrated (MCln·zH_2_O), where z represents the number of water molecules in the unit cell of salt. The general formula for this type of chemical is Cat + X^−^x(MCln)zH_2_O.
(iii) TYPE III: this subgroup is formed from the combination of quaternary ammonium salts such as HBA and HBD (carboxylic acid, alcohols, amides, carbohydrates, etc.).
(iv) TYPE IV: formed from transition metallic salts or hydrated metal salts and the corresponding hydrogen bond donor.
(v) TYPE V: in this class of DESs, both donors and acceptors of hydrogen bonds are non-ionic molecular substances, i.e., 1 thymol:2 menthol in molar ratio. Thus, they are not ionic, but they have the melting point characteristics presented by the other types of DEEs. This is attributable to the large number of hydrogen bonds present in this type V of DESs [20].

Apart from the above, a new type of eutectic solvents, i.e., natural deep eutectic solvents (NDESs), was reported [21], containing natural basic metabolites, including sugars, sugar alcohols, carboxylic acids, amino acids, and amines [22].

A novel DES formulation formed by dimethylthetin (DMT), oxalic acid dihydrate, and water, serving as chelating, reducing, and leaching agents, respectively, is used in the recycling of waste Li-ion batteries [23].

Following the preparation of new DESs, the preparation of DES based on imidazole and monoethanolamine is described [24]. 

Also, recently [25], the implications of using different approaches (no bond, generic bond, or single bond) to model the electrovalent or ionic interactions present in a hydrogen-bond acceptor molecule utilized in synthesizing DEEs have been reported. It is concluded that in the system formed by choline chloride and urea, the use of the above different approaches for modeling the ionic or electrovalent bonds in the acceptor molecules does not differentiate between the levels (PM3, HF, and DFT) of calculation utilized in the investigation. Moreover, the interaction of three H-atoms present in the alcohol functions of glycerol, as HBD, with the chloride ion of choline chloride, as HBA, is the thermodynamically feasible path for the choline chloride:glycerol compound formation.

Recently, the role of DESs (also ILs) in extractive metallurgy processes has been reviewed [26]. Despite the ample leadership given to these chemicals, at the present time (2024 year), there are still no commercial implantations using these green reactives. The above is attributed to a series of points, including (i) their low chemical stability under the working conditions of metallurgical processes, (ii) their high viscosity, which hindered phase disengagement (leading to dissolving them in traditional organic solvents), (iii) lack of pilot-scale demonstrations of proposed flow-sheets, and thus (iv) an interrogation about the cost of large-scale operations.

Within more or less the same opinion, it was stated that in liquid-liquid extraction DESs, there has not been any significant improvement in the use of conventional solvents [27].

In another review [28], the usefulness of DESs in extractive metallurgy is not as controversial as above, exploring a new generation of DESs and their uses in the actual research in this frontier area.

Despite all the controversy associated with these DESs, recent publications informed about the use of choline chloride-based DESs in recovering valuable metals (mainly lithium and cobalt) from spent batteries [29,30].

The present review described the most advanced data (2023 year) about using DESs to recover REEs. Moreover, the association between DESs and REEs in fabricating products for further use is described.

## 2. Utilization of DESs in the Recovery of REEs

### 2.1. DESs as Non-Aqueous Leachants of REEs

Monazite is one of the raw materials for REEs, and a process to dissolve this raw material using DESs as an environmental replacement for conventional acids (sulfuric and hydrochloric acids) was developed [31]. Various DESs (Table 1) were synthesized and utilized as non-aqueous leachant reagents of monazite.

Experimental results indicated that none of the above DESs recovered REEs (Ce, La, Nd, Dy, Eu, Gd, Pr, Sm) from the phosphate phase under the following conditions: 2 h at 120 °C and a solid/liquid ratio of 1. Thus, the monazite material was roasted in NaOH medium at 500 °C for two hours, and the resulting material was further leached with water. The roasting process transformed the phosphate salt into the corresponding hydroxide:

(1) $$\left(REE\right){PO}_{4}+3NaOH\to REE{\left(OH\right)}_{3}+{Na}_{3}{PO}_{4}$$

When the roasted process was performed at temperatures higher than 300 °C, the next reaction occurred:

(2) $$2REE{\left(OH\right)}_{3}\to {REE}_{2}O_{3}+3H_{2}O$$

The oxide was the final product. The roasted material was treated with the different DESs at 100 °C and for 72 h using a solid/liquid ratio of 10 g/L. The resulting data showed that with the ChCl:PTSA (2:1), ChCl:PTSA:EG (2:1:1), and PEG-400:PTSA (1:1) formulations, the highest extraction efficiencies (exceeding 80%) were obtained. No data about the recovery of the different REEs from the leach solution were given in the published manuscript.

### 2.2. DESs as a Medium to Separate REEs

#### 2.2.1. Electrodeposition

Apart from other characteristics, DESs have a wide electrochemical window that can be used in electrodeposition. Thus, a choline chloride-urea-based DES was proposed as a medium for electrodeposition of yttrium, samarium, and terbium [32]. All three metals were deposited in the DES using cyclic voltammetry and potentiostatic procedures. The results indicate that the electrodepositions of yttrium, samarium, and terbium in the DES were governed by diffusion, being the diffusion coefficients of yttrium, samarium, and terbium in DES at 60 °C of 7.37 × 10^−13^, 1.10 × 10^−12^, and 9.29 × 10^−13^ cm^2^/s, respectively. The three rare earth elements were deposited in the form of two-dimensional nanonetwork structures obtained through cyclic voltammetry or potentiostatic procedures.

A DES formed by betaine-ethylene glycol (Bet-EG) and Nd_2_O_3_ was utilized for the electrodeposition of the rare earth at various working temperatures in the 80–100 °C range [33]. The electrochemical behavior of Nd_2_O_3_ dissolved in Bet-EG DES was researched using cyclic voltammetry, potentiodynamic polarization, and chronoamperometry methods. Cyclic voltammograms (CVs) manifest that Nd(III) reduction in Bet-EG is an irreversible process with diffusion-controlled, and the reduction overpotential of Nd(III) decreases with the increase of the temperature. The temperature has an impact on the diffusion coefficient of Nd(III) species, obeying the Arrhenius equation. The activation energy is estimated to be 36.78 kJ mol^−1^. The cathodic polarization analysis shows that the rise of temperature promotes the reduction of Nd(III). The UV–Vis and ESI-MS analyses show that [Nd_2_(bet)_8_(H_2_O)_4_]^6+^ complex anions are formed as Nd_2_O_3_ is dissolved in Bet-EG DES. Electrodeposited neodymium with various morphologies was characterized and analyzed using EDX, XPS, XRD, and SEM. It was found that the electrodeposition temperature greatly affects the morphology of Nd electrodeposits. Depending on the deposition temperature, metallic Nd with submicron particles or porous structures were obtained.

#### 2.2.2. Membranes

This reference used an NDES formed by betaine and lactic acid (1:2 molar ratio) in the formulation of a polymer inclusion membrane to separate Nd, Sm, and Dy [34]. The preparation of the membrane also included the use of Cyanex 272 (phosphinic acid derivative) extractant. The use of NDES induced the pores on the surface to be much larger and abundant, and the migration of P-O and P[dbnd]O groups to the lower epidermal layer of the membrane assisted in increasing the hydrophilicity of the surface layer. The membrane containing 5 wt% NDES presented the best overall permeability and separation performances of the various formulations. Using EDTA (0.15 M) as the strippant or receiving phase, a maximum permeation flux of 4.5 µmol/m^2^·s was obtained, being the transport order Dy > Sm > Nd after 25 h. No data about how these REEs were separated from one another from the stripping phase were given in the manuscript; also, the role of Cyanex 272 in the membrane formulation was not mentioned.

#### 2.2.3. Solvent Extraction

A number of REEs (La, Ce, Pr, Nd, Eu, Gd, Y, and Lu) in the REEs^3+^ oxidation state plus Ce^4+^ were extracted using a mixture of decanoic acid and trioctylphosphine oxide under different experimental conditions: extractant formulation, nitric acid concentration, and temperature [35]. It was described that the REEs^3+^ were extracted using trioctylphosphine oxide dissolved in toluene by the next equilibrium:

(3) $${\left(REEs\right)}_{aq}^{3+}+3NO_{3}^{-}+3L_{org}↔(NO_{3}{)}_{3}\cdot 3L_{org}$$

where L represents the phosphine oxide and the subscripts aq and org are the respective aqueous and organic phases.

The various REEs were co-extracted (Table 2), though the extraction of the various elements was dependent on the aqueous phase acidity, and as a general rule, the extraction efficiency decreased at nitric acid concentrations in the 4–6 M range.

From the data presented in the above Table, it was clear that Ce(IV) was much better extracted by the decanoic acid+phosphine oxide formulation than the other REEs investigated in the present work. The manuscript did not give data about how the extracted REEs were stripped from the organic phase.

A DES based on trioctyl phosphine oxide and thenoyl trifluoroacetone was used in the extraction of europium and the actinides, UO_2_^2+^, Pu^4+^, and Am^3+^, from aqueous nitric acid medium [36]. At higher molarity of nitric acid (>5 M), the extraction becomes insignificant only for trivalent metal ions and opens up the possibility to selectively strip trivalent metal ions from tetravalent and hexavalent ions. This DES was also used for the dissolution of uranium oxide (UO_3_). The dissolution kinetics were studied, and it was shown that oxide was dissolved within an hour at 80 °C. The maximum solubility of UO_3_ in DES was measured and found to be 130 ± 5 mg/mL, which is one of the highest reported solubility of UO_3_ in ILs and DES. The species of uranium formed in situ in DES was ascertained to be UO_2_(TTA)_2_. TOPO after the dissolution of UO_3_ as supported by FTIR and NMR (1H and 31P) investigations.

2-hexyldecanoic acid and thymol were used to formulate DESs for the extraction of thorium from radioactive waste leach solution [37]. It was described that thorium extraction (exceeding 98%) was due to a cation exchange reaction with the acid:

(4) $${Th}_{aq}^{4+}+4HA_{org}↔ThA_{4}+4H_{aq}^{+}$$

where HA represented the organic acid molecule; thus, the extraction of the metal released protons in the raffinate. After adjusting the pH to 4, the authors claimed that this raffinate can be used to further recover the other REEs present in it. Thorium stripping was accomplished using a 0.2 M HCl solution. Over five cycles, there was no apparent loss of thorium extraction efficiency. Based on the separation factors (β_Th/M_, M = accompanying metal) values (Table 3), defined as the ratio of the distribution ratios:

(5) $$\beta_{Th/M}=\frac{D_{Th}}{D_{M}}$$

the system appeared to be highly selective with respect to thorium.

The next reference used a DES as a medium to purify Gd^3+^ by extracting the impurity (Al^3+^) present in GdCl_3_ solutions [38]. Using various formulations, Gd extraction was less than 20% in all the cases, whereas aluminum extraction exceeded 70% except in the case of the amide:genifibrozil (4:1) mixture. From these DESs, N,N-diethyldodecanamide::ibuprofen (3:1) formulation presented better separation performance, with a separation factor Al/Gd value greater than 400. Using a feed solution of near 854 mg/L Al(III) and 5.5 g/L Gd(III) and three continuous counter-current extraction stages, the experimental results showed that the above 3:1 formulation quantitatively extracted Al^3+^ (99%) from the feed GdCl_3_ solution. The rare earth element was slightly extracted (about 2.4%), resulting in a galdonymium raffinate of 99.9% purity. The extraction mechanism responsible for aluminum extraction was defined as an ion exchange mechanism; moreover, a saponification degree (0.03–0.085 mol/L) was beneficial in improving the extraction performance. The extraction circuit was closed with a scrubbing stage at pH 3 and aluminum stripping in an HCl medium. The system presented good stability over five continuous extraction-scrubbing-stripping cycles.

The solvent extraction of Am(III) and Eu(III) using DESs was modeled [39]. In this case, DESs formed by TOPO:oxalic acid and TOPO:maleic acid as HBA:HBD in a 1:1 molar ratio were used to extract Am(III) from nitric acid media. In the extraction of Am(III) and Eu(III), a DES formed by choline acetate:glycolic acid (as HBA:HBD also in 1:1 molar ratio) was used as an organic medium of the extractant phase formed by CyMe_4^−^_BTBP (6,6′-bis(5,5,8,8-tetramethyl-5,6,7,8-tetrahydrobenzo-1,2,4-triazin-3-yl)-2,2′-bypyridine)) in 1-octanol. The model reasonably predicted the experimental data for the systems involving the extraction of Am(III) with TOPO:organic acids. However, severe deviations occurred in the systems involving the extraction of Am(III) and Eu(III) in choline acetate:glycolic acid medium. Moreover, the DES was solubilized in the raffinate in these last two systems.

The quaternary ammonium salt Aliquat 336 and glycerol mixture diluted in kerosene was used to investigate Nd/Fe and Sm/Co separation from nitrate medium [40]. In the Nd/Fe system, the rare earth (near 94% extraction) was separated from iron(III) under the following experimental conditions: organic to aqueous phase volume ratio of 2, pH 2, extractant concentration of 0.2 M, 25 °C, with the feed solution also containing 3.5 M sodium nitrate. In the case of the Sm/Co system, samarium was extracted best (near 94%) under an O/A phases ratio of 4, pH 2, 0.2 M DES, and 25 °C; in this case, the presence of the nitrate salt improved the metal extraction. Quantitative Nd(III) or Sm(III) was achieved using 0.6 M HCl. It should be noted that though iron(III) was slightly extracted, cobalt(II) was not extracted in any experimental condition. This reference demonstrated that the use of an organic diluent was necessary to perform metal extraction. Moreover, the manuscript presented data on how the variation in the diluent influenced the REE extraction (Table 4).

Also, note the use of a forbidden diluent (carbon tetrachloride) due to cancer issues. Reviewers and Editors of this manuscript should never have allowed the publication of the data relative to the use of this harmful diluent.

Tri-octylphosphine oxide and isostearic acid (1:1 molar ratio) formed a DES utilized to investigate its performance in the extraction of scandium in the presence of yttrium, iron, and aluminum [41]. The extractant was diluted in toluene, and scandium was extracted into the organic phase by the next equilibrium:

(6) $${Sc}_{aq}^{3+}+2.5{\left(HA\right)}_{2org}+5L_{org}↔Sc{\left(HA\right)}_{2}A_{3}\cdot 5L_{org}+3H_{aq}^{+}$$

where HA represents the acid molecule, and L is the phosphine oxide molecule. Thus, the extraction responded to a cation exchange mechanism, in which one mol of scandium extracted to the organic phase released three mol of protons to the raffinate. Accordingly, the stripping reaction occurred by shifting the equilibrium to the left, and scandium can be stripped by using 2 M HCl or sulfuric acid solutions. Though the authors claimed that the extractant can separate scandium from the other elements present in the feed solution, the manuscript did not present clear data about these separations. Also, the manuscript did not contemplate the recovery of scandium from these highly acidic stripping solutions. Lastly, this investigation is another example of the use of an ordinary diluent to dissolve DESs.

## 3. Miscellaneous DESs and REEs Uses

A DES, formed by tetra-butyl phosphoniumbromide and various organic acids as hydrogen bond donors, was coated with cerium oxide nanoparticles to investigate its performance on CO_2_ capture [42]. The DES formed by the phosphonium salt and formic acid (1:1 molar ratio) presented the best CO_2_ uptake of 0.056 mmol/g, whereas the DES containing butyric acid was the formulation with the lower CO_2_ capture (0.041 mmol/g). The better CO_2_ capture of the phosphonium salt:formic acid formulation was attributable to the high density of carboxylic functional groups, which improved the physisorption capture process due to the enrichment of binding energies. The manuscript did not present data about the recyclability of the adsorbent.

Choline chloride and urea in a 1:2 molar ratio formed a DES utilized in the synthesis of praseodymium vanadate nanoparticles [43]. These nanoparticles were fabricated by a solvothermal procedure using praseodymium nitrate and ammonium vanadate dissolved in the above DES as precursors. The PrVO_4_ nanoparticles were used as an electrochemical sensor for furaltadone (FLD) detection. A maximum in the peak current occurred when the pH of the electrolyte solution reached the value of 7 (Table 5).

During the furaltadone reduction process, the amount of FLD^+^ in the electrolyte increased, resulting in a decrease of FLD^−^ species. Thus, a maximum in the current is reached due to the influence of hydrogen bonding and electrostatic interactions. Moreover, at this pH of 7, the PrVO_4_/GCE nanocomposite has greater electron mobility due to its contributed excited electrons.

Various DESs were fabricated using choline chloride and different salts of various REES (lanthanum, cerium, europium, and samarium) in the 1:0.5–1.3 ratio [44]. Further, these mixtures were used to form the vanadium phosphorous oxide (VPO) catalyst used to investigate its catalytic performance in n-butane selective oxidation to produce maleic anhydride. The presence of the REE-DESs tuned the structural modifiers and electronic promoters during the synthesis of the catalyst and, thus, tuned the physicochemical properties of the VPO catalysts. Though the different REE-DES improved n-butane conversion and MA selectivity, the Ce-DES-VPO formulation presented the best results with respect to the above points of conversion and selectivity.

Another DES, formed by cetyltrimethylammonium bromide:urea:glycerol in the molar ratio 1:2:5 together with yttrium nitrate hexahydrate and WO_3_ nanoparticles, was used to fabricate a WO_3_:Y_2_O_3_ nanocomposite [45]. The use of the DES promoted changes in the nanocomposite’s porous nature, size, and morphology. Using this nanocomposite, it was shown that the frequency-dependent AC and DC conductivities were temperature-dependent, increasing with the increase of this variable from 30 °C to 150 °C. From the GCD curves, it was observed that the highest capacitance of 460 Fg^−1^ was obtained at a current density of 2 Ag^−1^, and cycling stability was found to be around 79% for up to 3050 cycles.

Different complexes of cerium(III) salt dissolved in a DES formed by choline chloride, urea, and water, with different molar hydration ratios (w) of 2, 5, and 10, were measured using neutron diffraction with isotopic substitution, and the various structures were modeled using empirical potential structure refinement (EPSR) [46]. These various structures depended on the molar hydration ratio presented by the DES (Table 6).

This rare earth element formed highly charged complexes with coordination numbers of 7–8, in which the shell contained chloride and water. Cluster information highlighted the trace presence of percolating water clusters (25 ≥ n ≥ 2) in 5 w and 10 w DES formulations.

The self-aggregation process of three surfactants, anionic sodium dodecylsulfate (SDS), cationic cetyltrimethylammonium bromide (CTAB), and non-ionic Triton X-100 (TX-100), dissolved in DESs composed of a lanthanide salt (Ln = La(III) or Ce(III)) and urea was investigated [47]. The self-assembly process was comparable to that in water, being energetically favorable. The type of lanthanide element did not affect the aggregation efficiency; however, the concentration of urea in the DES did. The increase of urea in the DES decreased the self-aggregation of both anionic and cationic surfactants. This was attributable to the different thermodynamic parameters involved in the aggregation process. This type of aggregation using these DESs may improve the applications of these systems in several fields: material synthesis, nanoreactors/nanocarriers, etc.

A DES formulated with heptyltriphenylphosphonium bromide:decanoic acid (1:2 ratio) and europium(III) was used to investigate the luminescent and electrochemical properties [48]. The Eu–DES complex was formed by the following reaction:

(7) $$[Eu{(H}_{2}O{)}_{8}{]}^{3+}+2{NO}_{3}^{-}+DES\to [Eu\cdot DES\cdot {(NO}_{3}{)}_{2}{]}^{+}+H_{2}O$$

The electrochemical results showed that the redox reaction of Eu(III)/Eu(II) in the DES has a quasi-reversible behavior and that the reaction rate increased with higher temperatures.

Electrolytes based on a mixture of choline chloride and ethylene glycol, both forming a DES nicknamed ethylene, containing dissolved LaCl3 and NiCl2, were used as a source for the electrodeposition of Ni-La coatings [49]. The presence of lanthanum in the nickel matrix served to increase the electrocatalytic activity due to (i) the presence of lanthanum in the (II) and (III) oxidation states and (ii) the synergistic interaction between both metals. These types of materials will probably be of utility in the production of green hydrogen through electrolytic procedures.

Very close to the previous reference, an electrolyte based on a DES formed by a mixture of choline chloride and urea, named reline, was used to electrodeposition coatings containing Ni-Ce [50]. An increase in the electrocatalytic activity was found to occur when the concentration of cerium in the coating increased. In systems containing cerium(III), reline-based electrolytes formed coatings with greater activity towards hydrogen evolution reaction than coatings formed by utilizing electrolytes based in the DES formed by choline chloride and ethylene glycol.

A 1:2 M solution of choline chloride and ethylene glycol formed a DES from which LaF_3_ was added in order to deposit the lanthanum salt on the pore walls of porous silicon and investigate the photoluminescence properties of this LaF_3_-passivated porous silicon structure [51]. In the synthesis of the final material, firstly, the LaF_3_-DES phase was spin-coating deposited on the pore wall of the porous silicon and annealed to evaporate the DES, leaving the lanthanum salt on the pore wall, forming a passivating layer. Experimental results indicated that the passivated material presented a higher luminescence than the pristine porous silicon; these results were attributable to a unique chemical process involving the DES and that the regulation of the spin coater speed improved this process.

Mixed matrix membranes used to investigate the permeation of CO_2_ were fabricated from ceria nanoparticles-DES, which acted as a filler for the membrane [52]. The DES used in the investigation was formed by cetrimonium bromide and acetic acid in a 1:1 ratio. Further to the casting process, it was demonstrated that the filler was dispersed in a uniform form in the polysulfone and that the polymer and the ceria-DES filler did not react between them. Experimental results indicated that the mixed matrix membrane performed better than the pristine polysulfone membrane with respect to CO_2_ permeation (Table 7). Also, CO_2_ selectivity against the presence of CH_4_ or N_2_ in the gas stream was improved using the present mixed matrix membranes.

## 4. Conclusions

Deep eutectic solvents are used to recover REEs from different sources, both to dissolve them from the different solids containing them and in the separation operations to recover pure products. Thus, solvent extraction operations are presented in most publications related to these separations.

These applications and the term green attached to the name of DESs sometimes became overshadowed by the utilization of traditional organic diluents, most probably due to the high viscosity presented from DESs, which increases as these chemicals became loaded by REEs (metals in general), making these processes not as environmentally as they appear to be. It is also striking that some authors used carcinogenic diluents (i.e., CCl_4_) in their experimentations.

Apart from the limitations of viscosity, another odd feature of using DESs in these separations systems is that, in some of the proposed processes of REE recovery, the regeneration of DESs is not fully understood and is even unexplained or unresolved.

Though the future of using DESs in leaching and separation operations is still open, some limitations must be resolved, i.e., loss of the cationic moiety to the aqueous phase and the own solubilization of DESs in water. These resolutions must be performed first at the laboratory scale, before scaling up to a pilot plant or demonstration plant, and before it becomes an industrial process, which seems to be a chimera.

It is of positive interest to note how DESs are used as a medium or take part in the fabrication of materials involving REEs, with a wide field of applications in different disciplines. 

## Figures and Tables

**Table 1:** DES formulations used in the dissolution of monazite.

HBA	HBD	Molar Ratio HBA:HBD
PEG-400	PTSA	1:1
Choline chloride	PTSA	2:1
Choline chloride	Urea	1:2
Choline chloride	EG	1:2
Choline chloride	Urea:EG	2:4:1
NaOH	PEG-200	1:44
NaOH	PEG-400	1:44
Choline chloride	PTSA:EG	2:1:1
Choline chloride	PTSA:PEG	2:1:1
Choline chloride	AA	1:2
Choline chloride	DLLA	1:2

PEG: polyethylene glycol. PTSA: p-toluenesulfonic acid. EG: ethylene glycol. AA: acetic acid. DLLA: DL-lactic acid.

**Table 2:** Distribution ratios of the various REEs at different nitric acid concentrations.

Element	0.1 M HNO_3_	1 M HNO_3_	6 M HNO_3_
La(III)	0.59	1.0	0.19
Ce(III)	0.63	1.90	0.05
Ce(IV)	12.0	11.0	6.52
Pr(III)	2.77	3.47	0.02
Nd(III)	1.49	3.16	0.19
Eu(III)	6.56	8.16	0.51
Gd(III)	3.93	4.94	0.36
Y(III)	1.60	6.38	1.18
Lu(III)	2.98	6.72	1.90

**Table 3:** Separation factors in the extraction of Th(IV) from other elements present in the waste leachate.

La(III): 1.9 × 10^5^	Gd(III): 2.5 × 10^4^	Tm(III): 2.6 × 10^4^
Ce(III): 1.2 × 10^5^	Tb(III): 1.7 × 10^4^	Yb(III): 2.5 × 10^4^
Pr(III): 5.5 × 10^4^	Dy(III): 2.3 × 10^4^	Lu(III): 1.4 × 10^4^
Nd(III): 5.1 × 10^4^	Ho(III): 2.5 × 10^4^	Mg(II): 2.9 × 10^6^
Sm(III): 8.7 × 10^3^	Y(III): 5.3 × 10^4^	Al(III): 1.5 × 10^5^
Eu(III). 1.9 × 10^4^	Er(III): 2.8 × 10^4^	Ca(II): 1.3 × 10^6^

**Table 4:** Approximate distribution ratio values using various diluents.

Diluent	Nd(III)	Fe(III)	Sm(III)	Co(II)
Heptane	20	<0.5	1.5	No extraction
Hexane	3	<0.5	1.75	No extraction
Carbon tetrachloride	5	<0.5	2	No extraction
Kerosene	6.5	0.5	2.5	No extraction

**Table 5:** Influence of the electrolyte pH on current.

pH	Current, µA
3	−16
5	−21
7	−24
9	−16

**Table 6:** Cerium(III) complexes as a function of the molar hydration ratio of the DES.

Molar Hydration Ratio	Complex
2 w	[CeCl_6_H_2_O]^3−^
5 w	[CeCl_5_(H_2_O)_2_]^2−^
10 w	[CeCl_5_(H_2_O)_3_]^2−^

**Table 7:** CO_2_ permeation (Barrer units) using both types of membranes.

	CO_2_ Pure	CO_2_/CH_4_	CO_2_/N_2_
Pristine membrane	6.7	6.06	6.36
Mixed matrix membrane	17.2	16.3	16.9

## Data Availability

Data are contained within the article.

## References

[1] US Geological Survey, Statista, 2023. https://www.statista.com.

[2] Investing News Network. https://investingnews.com.

[3] Liu S.-L., Fan H.-R., Liu X., Meng J., Butcher A.R., Yann L., Yang K.-F., Li X.-C. (2023). Global rare earth elements projects: New developments and supply chains. Ore Geol. Rev..

[4] Ray A.R., Mishra S. (2023). Hydrometallurgical technique as better option for the recovery of rare earths from mine tailings and industrial wastes. Sustain. Chem. Pharm..

[5] Han K.N., Kim R., Kim J. (2023). Recent Advancements in Hydrometallurgy: Solubility and Separation. Trans. Indian Inst. Met..

[6] Abbott A.P., Boothby D., Capper G., Davies D.L., Rasheed R.K. (2004). Deep eutectic solvents formed between choline chloride and carboxylic acids: Versatile alternatives to ionic liquids. J. Am. Chem. Soc..

[7] Hansen B.B., Spittle S., Chen B., Poe D., Zhang Y., Klein J.M., Horton A., Adhikari L., Zelovich T., Doherty B.W. (2021). Deep eutectic solvents: A review of fundamentals and applications. Chem. Rev..

[8] El Achkar T., Greige-Gerges H., Fourmentin S. (2023). Basics and properties of deep eutectic solvents: A review. Environ. Chem. Lett..

[9] Cichowska-Kopczynska I., Nowosielski B., Warminska D. (2023). Deep eutectic solvents: Properties and applications in CO_2_ separation. Molecules.

[10] Prabhune A., Dey R. (2023). Green and sustainable solvents of the future: Deep eutectic solvents. J. Molec. Liq..

[11] Chen Y., Mu T. (2021). Revisiting greenness of ionic liquids and deep eutectic solvents. Green Chem. Eng..

[12] Suthar P., Kaushal M., Vaidya D., Thakur M., Chauhan P., Angmo D., Kashyap S., Negi N. (2023). Deep eutectic solvents (DES): An update on the applications in food sectors. J. Agric. Food Res..

[13] Atilhan M., Aparicio S. (2022). Molecular dynamics study on the use of deep eutectic solvents for enhanced oil recovery. J. Pet. Sci. Eng..

[14] Liu Z., Zhao G., Brewer M., Lv Q., Sudholter E.J.R. (2021). Comprehensive review on surfactant adsorption on mineral surfaces in chemical enhanced oil recovery. Adv. Colloid Interface Sci..

[15] Abbott A.P., Capper G., Davies D.L., Munro H.L., Rasheed R.K., Tambyrajah V. (2002). Preparation of novel, moisture-stable, Lewis-acidic ionic liquids containing quaternary ammonium salts with functional side chains. Chem. Commun..

[16] Abbott A.P., Capper G., Davies D.L., Rasheed R.K., Tambyrajah V. (2002). Quaternary ammonium zinc- or tin-containing ionic liquids: Water insensitive, recyclable catalysts for Diels—Alder reactions. Green Chem..

[17] Abbott A.P., Capper G., Davies D.L., Rasheeda R.K., Tambyrajaha V. (2003). Novel solvent properties of choline chloride/urea mixtures. Chem. Commun..

[18] Shirota H., Koyakkat M., Cao M., Shimizu M., Asakura S., Kawamoto H., Moriyama K. (2023). Facile preparation of deep eutectic solvents having high electrical conductivities. J. Molec. Liq..

[19] Koyakkat M., Moriyama K., Asakura S., Kawamoto H., Shirota H. (2023). Deep eutectic solvents based on ammonium iodide and iodine possessing high electrical conductivity. J. Molec. Liq..

[20] Omar K.A., Sadeghi R. (2023). Database of deep eutectic solvents and their physical properties: A review. J. Mol. Liq..

[21] Choi Y.H., van Spronsen J., Dai Y., Verberne M., Hollmann F., Arends I.W.C.E., Witkamp G.J., Verpoorte R. (2011). Are natural deep eutectic solvents the missing link in understanding cellular metabolism and physiology?. Plant Physiol..

[22] Długosz O. (2023). Natural deep eutectic solvents in the synthesis of inorganic nanoparticles. Materials.

[23] Luo Y., Yin C., Ou L. (2023). Recycling of waste lithium-ion batteries via a one-step process using a novel deep eutectic solvent. Sci. Total Environ..

[24] Mjalli F.S., Shakourian-Fard M., Kamath G., Murshid G., Naser J., Al Ma’awali S. (2023). Experimental and theoretical study of the physicochemical properties of the novel imidazole-based eutectic solvent. J. Mol. Graph. Model..

[25] Uzochukwu M.I., Oyegoke T., Momoh R.O., Isa M.T., Shuwa S.M., Jibril B.Y. (2023). Computational insights into deep eutectic solvent design: Modeling interactions and thermodynamic feasibility using choline chloride & glycerol. Chem. Eng. J. Adv..

[26] Binnemans K., Jones P.T. (2023). Ionic liquids and deep-eutectic solvents in extractive metallurgy: Mismatch between academic research and industrial applicability. J. Sustain. Metal..

[27] Kanzaki R. (2023). Deep eutectic solvents for liquid-liquid extraction. Anal. Sci..

[28] Panda P., Mishra S. (2023). Deep eutectic solvents: Physico-chemical properties and their use for recovery of metal values from waste products. J. Mol. Liq..

[29] Wang M., Liu K., Xu Z., Dutta S., Valix M., Alessi D.S., Huang L., Zimmerman J.B., Tsang D.C.W. (2023). Selective extraction of critical metals from spent lithium-ion batteries. Environ. Sci. Technol..

[30] Zhu A., Bian X., Han W., Wen Y., Ye K., Wang G., Yan J., Cao D., Zhu K., Wang S. (2023). Microwave-ultra-fast recovery of valuable metals from spent lithium-ion batteries by deep eutectic solvents. Waste Manag..

[31] Shakiba G., Saneie R., Abdollahi H., Ebrahimi E., Rezaei A., Mohammadkhani M. (2023). Application of deep eutectic solvents (DESs) as a green lixiviant for extraction of rare earth elements from caustic-treated monazite concentrate. J. Environ. Chem. Eng..

[32] Jiang X., Chen C., Huang D., Zhao X., Wei L. (2023). Electrodeposition of rare-earth metals (yttrium, samarium and terbium) from a deep eutectic solvent. Xiyou Jinshu Cailiao Yu Gongcheng/Rare Met. Mater. Eng..

[33] Xiang G., Xu C., Wang S., Li J., Chen W., Gu D., Zhang Q., Hua Y. (2023). Electrodeposition of neodymium from betaine-ethylene glycol deep eutectic solvent using neodymium oxide as a precursor. Electrochem. Commun..

[34] Chen L., Cui R., Pan W., Dai J., Meng M., Dai X., Pan J. (2023). Role of natural deep eutectic solvents (NADESs) in coagulation bath for PVDF-based membranes on enhanced permeation and separation of rare earth ions. J. Membr. Sci..

[35] Favero U.G., Schaeffer N., Passos H., Cruz K.A.M.L., Ananias D., Dourdain S., Hespanhol M.C. (2023). Solvent extraction in non-ideal eutectic solvents–Application towards lanthanide separation. Sep. Purif. Technol..

[36] Gamare J., Vats B.G. (2023). A hydrophobic deep eutectic solvent for nuclear fuel cycle: Extraction of actinides and dissolution of uranium oxide. Eur. J. Inorg. Chem..

[37] Ni S., Gao Y., Yu G., Zhang S., Zeng Z., Sun X. (2023). A sustainable strategy for targeted extraction of thorium from radioactive waste leachate based on hydrophobic deep eutectic solvent. J. Hazard. Mater..

[38] Ni S., Yu G., Gao Y., Zhang S., Su H., Sun X. (2023). Tailored hydrophobic deep eutectic solvent for removing trace aluminum impurity to produce high-purity GdCl3. Sep. Purif. Technol..

[39] Patra D.K., Thombre A.V., Kundu D. (2023). Generalized Pitzer-Debye-Hückel (PDH) framework for the deep eutectic solvent assisted extraction of europium (III), americium (III), and uranium (VI). Solvent Extr. Ion Exch..

[40] Prusty S., Pradhan S., Mishra S. (2023). Extraction and separation studies of Nd/Fe and Sm/Co by deep eutectic solvent containing Aliquat 336 and glycerol. J. Chem. Technol. Biotechnol..

[41] Ushizaki S., Kanemaru S., Sugamoto K., Baba Y. (2023). Selective extraction equilibria of Sc(III), Y(III), Fe(III) and Al(III) from acidic media with toluene mixture of deep eutectic solvent (DES) composed of TOPO and isostearic acid. Anal. Sci..

[42] Ahmad T., Iqbal J., Bustam M.A., Babar M., Tahir M.B., Sagir M., Irfan M., Asghar H.M.A., Hassan A., Riaz A. (2023). Performance evaluation of phosphonium based deep eutectic solvents coated cerium oxide nanoparticles for CO_2_ capture. Environ. Res..

[43] Chen T.-W., Priya T.S., Chen S.-M., Kokulnathan T., Ahmed F., Alshahrani T. (2023). Synthesis of praseodymium vanadate in deep eutectic solvent medium for electrochemical detection of furaltadone. Process Saf. Environ. Prot..

[44] Faizan M., Li Y., Wang X., Song P., Zhang R., Liu R. (2023). Rare earth metal based DES assisted the VPO synthesis for n-butane selective oxidation toward maleic anhydride. Green Energy Environ..

[45] Joel C., Bennie R.B., Antony A.J., Abi S.V.V. (2023). Role of deep eutectic solvent in the surface modification of yttria based WO_3_ nanocomposite for application in nanoarchitectonics. Ceram. Int..

[46] Hammond O.S., Bathke E.K., Bowron D.T., Edler K.J. (2023). Trace water changes metal ion speciation in deep eutectic solvents: Ce^3+^ solvation and nanoscale water clustering in Choline Chloride—Urea—Water Mixtures. Inorg. Chem..

[47] Khokhar V., Anjali, Pandey S. (2023). Constituent- and composition-dependent surfactant aggregation in (lanthanide salt + urea) deep eutectic solvents. Langmuir.

[48] Patil S.M., Agrawal R., Gupta R., Gupta S.K., Ghosh A., Kumar S., Jayachandran K., Ghanty T.K. (2023). Understanding the excited state dynamics and redox behavior of highly luminescent and electrochemically active Eu(III)–DES complex. Dalton Trans..

[49] Protsenko V.S., Pavlenko L.M., Bobrova L.S., Korniy S.A., Butyrina T.E., Danilov F.I. (2023). Ni-La coatings as electrocatalysts for hydrogen evolution reaction deposited from electrolytes based on a deep eutectic solvent. Vopr. Khimii i Khimicheskoi Tekhnologii.

[50] Protsenko V.S., Pavlenko L.M., Bobrova L.S., Korniy S.A., Danilov F.I. (2023). Electrodeposition of coatings from urea–choline chloride-based plating baths containing Ni(II) and Ce(III) chloride salts and electrocatalytic activity of electrodeposits towards the hydrogen evolution reaction. J. Solid State Electrochem..

[51] Rahman M.H., Ahmed S., Mou S.S., Ismail A.B. (2023). Efficient passivation of porous silicon with LaF3 by deep eutectic solvent based novel chemical route. Mater. Sci. Eng. B.

[52] Saif-ur-Rehman M., Mehdi M.S., Fakhar-e-Alam M., Asif M., Rehman J., Alshgari R.A., Jamal M., Zaman S.U., Umar M., Rafiq S. (2023). Deep eutectic solvent coated cerium oxide nanoparticles based polysulfone membrane to mitigate environmental toxicology. Molecules.

